# Nonunion After First Metatarsophalangeal Joint Arthrodesis: The Association With Shoe Size

**DOI:** 10.7759/cureus.61448

**Published:** 2024-05-31

**Authors:** Wout Füssenich, Martin Stevens, Julian R Zwoferink, Jessie M. M Schoenaker, Matthijs P Somford, Gesine H Seeber

**Affiliations:** 1 Department of Orthopedic Surgery, University of Groningen, University Medical Center Groningen, Groningen, NLD; 2 Faculty for Human Movement Sciences, University of Groningen, University Medical Center Groningen, Groningen, NLD; 3 Department of Orthopedic Surgery, Rijnstate Hospital, Arnhem, NLD; 4 School of Medicine and Health Sciences, Division of Orthopedics at Campus Pius-Hospital, Carl von Ossietzky Universität Oldenburg, Oldenburg, DEU

**Keywords:** hallux, shoe size, first metatarsophalangeal joint, fusion, arthrodesis

## Abstract

Introduction: First metatarsophalangeal joint (MTPJ) arthrodesis is a common treatment for various foot conditions, with nonunion as a frequent complication. The incidence of nonunion varies widely in the literature. In particular, males have a higher risk of nonunion than females. This is possibly due to biomechanical and anatomical differences, as men have on average larger feet than women. This study therefore aims to explore whether shoe size, as a proxy for foot size, affects nonunion rates and could explain the gender disparity in nonunion rates.

Methodology: An exploratory analysis of retrospectively collected data from patients who underwent primary first MTPJ arthrodesis in a single secondary hospital between January 2012 and December 2019. Additional data on body weight, height, and shoe size were prospectively collected from patients.

Results: Among 261 included patients, 57 (21.8%) experienced nonunion. Nonunion incidence was higher in males (18, 26.9%) than in females (39, 20.1%). Self-reported shoe size showed no significant association with nonunion in both univariate and multivariate analyses.

Discussion: The study’s findings suggest that shoe size, as a proxy for foot size, is not associated with nonunion after the first MTPJ arthrodesis. Despite observing a gender difference in nonunion rates, this disparity could not be explained by shoe size.

Conclusions: Shoe size as a proxy for foot size appears to have no clinical association with nonunion following the first MTPJ arthrodesis.

## Introduction

First metatarsophalangeal joint (MTPJ) arthrodesis is a widely used treatment for moderate to severe hallux valgus, symptomatic hallux rigidus, and failed hallux valgus correction [1]. A common complication of arthrodesis is nonunion [2]. The range of nonunion incidence in literature is 0% to 12% [2-5]. However, results from our own research suggest an even higher incidence of 15.2% [6]. The widespread incidence reported could be explained by a large heterogeneity of pathologies and surgical techniques used [2,5-7]. Several other nonunion risk factors have been described, such as high preoperative and postoperative hallux valgus [6,8-10], smoking [11,12], and comorbidities like diabetes [8] and rheumatoid arthritis [3]. Interestingly, the male sex as a risk factor for nonunion after the first MTPJ arthrodesis is also reported [13-16]. Reported incidences vary for men between 13.9% and 17.5% and women between 3.8% and 5.8%. In the results of our own study, we saw a similar pattern with 19.5% nonunion in males versus 13.6% in females, which is even higher compared to earlier literature [6].

It can therefore be questioned where this difference between men and women comes from. Bass and Sirikonda hypothesized that the difference between sexes is due to men’s lower adherence to postoperative instructions, as they saw greater differences in nonlocking constructions [13]. However, biomechanical aspects can also play a role since Wunderlich and Cavanagh demonstrated that female feet differ in several shape characteristics, particularly at the arch, lateral side of the foot, first toe, and ball of the foot [17]. Putti et al. showed higher pressure forces under the first metatarsal during gait in males compared to females, which might be a result of these anatomical differences, higher body weight and height, and stiffer joints in the foot [18]. In addition, men on average have larger feet than women [17,19,20]. Increased size of metatarsals and phalanges results in greater force vectors exerted on the joints [21].

The physical differences between males and females can ultimately impose greater stress on the fixation material in men and potentially result in implant failure, leading to nonunion [22,23]. Numerous investigations have assessed the impact of body mass index (BMI), body weight, and height, but the influence of foot size remains unexplored in the existing literature. It can thus be questioned whether foot size affects the occurrence of nonunion. The aim of this study is, therefore, to explore whether shoe size as a proxy of foot size affects the occurrence of nonunion following the first MTPJ arthrodesis and could explain the difference in nonunion between men and women.

## Materials and methods

An exploratory analysis based on retrospectively gathered data. All patients who underwent primary first MTPJ arthrodesis at the secondary care hospital Rijnstate, The Netherlands, between January 2012 and December 2019 and gave written informed consent were included. Data were recorded from medical records. Patients were prospectively approached at least one year after surgery, with an information letter and asked about their body weight and height, as well as their shoe size. Patients were called when they didn’t respond to the information letter. Two unanswered telephone calls led to patient exclusion. The local ethics committee of Rijnstate Hospital approved the study before initiation (registration no. 2021-1975) on January 31, 2021.

Measures

Dependent Variables

The incidence of the first MTPJ nonunion was the primary outcome. Nonunion was defined as the absence of radiological signs of bridging and/or hardware failure (radiolucency/osteolysis, broken hardware, or migration) after at least six months of follow-up. The distinction between symptomatic versus asymptomatic nonunion was made by the presence of pain and/or loss of function with radiological evidence of nonunion.

Independent Variables

Self-reported shoe size served as the independent variable. Patients reported the shoe size they regularly wear according to the European size convention.

Covariates

Anthropometric data (sex, age, body height and weight, and BMI), operated side (right/left), American Society of Anesthesiologists (ASA) classification (categorized as ≤ II and ≥III), postoperative hallux valgus angle (HVA), type of any revision surgery, and wound infection or wound healing disorders (yes/no) were registered. The latter was defined as wound dehiscence or signs of inflammation two weeks postoperatively. Articular surface preparation methods (planar cuts, convex/concave reamers, or manual preparation with hand instruments), fixation methods (a plate combined with an interfragmentary screw (IFS), crossed screws, or only a plate); type of postoperative immobilization (six weeks of hallux cast/foot cast combined with forefoot off-load shoes, six weeks of short leg cast combined with forefoot off-load shoes, and 8-12 weeks of hallux cast/foot cast combined with forefoot off-load shoes), and pre- and postoperative HVA.

Statistical Analysis 

All data were processed in SPSS (IBM SPSS v28.0 Inc. Chicago, IL) for statistical analysis. Descriptive statistics were used to describe sample characteristics. Normally distributed data are presented as mean with standard deviation, and non-normally distributed data are presented as median with interquartile range. Categorical data are presented as a number with a corresponding percentage.

Univariate logistic regression analysis was performed to identify differences between the first MTPJ union and nonunion, with union as the reference category. Subsequently, in addition to shoe size, all covariates with *P *< 0.157 were entered in a multiple regression analysis [24,25]. After checking for multicollinearity (variance inflation factor [VIF] < 5.0) [26], stepwise regression procedures were used to evaluate the significance and fit statistics (‑2 log-likelihood). As a secondary analysis, postoperative HVA was included in the model. The results were reported as odds ratios (OR) with 95% confidence intervals (95% CIs). Continuous variables were also presented with the nonstandardized regression coefficient (log odds). Differences were considered statistically significant with a *P*-value < 0.05.

## Results

Sample characteristics

In total, 345 patients underwent primary first MTPJ arthrodesis in the set period. The municipal registry was consulted for information on deceased patients. Seventeen patients had died and were, therefore, excluded. Five patients moved without a forwarding address, so the information letters were returned. Six patients did not want to participate. The remaining 56 patients did not respond to the information letter or two telephone attempts. Hence, in total, 261 patients signed informed consent and were included.

Of the included participants, 194 were female and 67 were male. The mean age was 60, and the mean BMI was 27.5. The total mean preoperative HVA was 27°. Nonunion was found in 57 out of 261 patients (21.8%). Demographic characteristics are presented in Table 1. 

**Table 1:** Sample characteristics stratified for first MTPJ union and nonunion. ^*^Continuous variables are presented: mean (standard deviation). Variables presented as *n*, *n* (%), or mean (SD) SD, standard deviation; ASA, American Society of Anesthesiologists classification; BMI, body mass index; CC, convex/concave; IFS, interfragmentary screw

Variable	Total (*n* = 261)	Union (*n* = 204)	Nonunion (*n* = 57)
Age (years)*	60.33 (10.2)	60.5 (10.4)	59.7 (9.4)
Sex, *n* (%)			
Female	194 (74.3%)	155 (79.9%)	39 (20.1%)
Male	67 (25.7%)	49 (73.1%)	18 (26.9%)
ASA (%)			
≤II	238 (91.2%)	184 (60.0%)	54 (40.0%)
≥III	23 (8.8%)	20 (87.0%)	3 (13.0%)
Side, *n* (%)			
Right	125 (47.9%)	102 (81.6%)	23 (18.4%)
Left	136 (52.1%)	102 (75.0%)	34 (25.0%)
BMI (kg/m^2^)*	27.5 (4.9)	27.3 (4.9)	28.2 (4.8)
Body weight (kg)*	79.5 (16.8)	78.6 (16.6)	82.7 (17.5)
Body height (cm)*	169.7 (8.9)	169.4 (8.9)	170.9 (9.1)
Active smoker, *n* (%)			
Yes	9 (3.4%)	6 (66.7%)	3 (33.3%)
No	73 (27.9%)	52 (71.2%)	21 (28.8%)
Missing	179 (68.6%)		
Wound infection, *n* (%)			
Yes	13 (4.9%)	9 (69.2%)	4(30.8%)
No	248 (95%)	195 (78.6%)	53 (21.4%)
Additional forefoot surgery			
Yes	41 (15.7%)	31 (75.6%)	10 (24.4%)
No	220 (84.3%)	173 (78.6%)	47 (21.4%)
Hallux valgus angle (°)			
Preoperative*	27.0 (14.0)	25.8 (13.5)	31.4 (14.6)
Postoperative*	16.9 (8.5)	16.2 (8.0)	19.4 (9.7)
Joint preparation technique			
Planar cuts	24 (9.2%)	22 (91.7%)	2 (8.3%)
CC reaming	135 (51.7%)	106 (78.5%)	29 (21.5%)
Manual preparation	102 (39%)	76 (74.5%)	26 (25.5%)
Joint fixation technique			
Plate + IFS	5 (1.9%)	4 (80.0%)	1 (20.0%)
Crossed screws	238 (91.2%)	186 (78.2%)	52 (21.8%)
Plate only	15 (5.7%)	12 (80.0%)	3 (20.0%)
Postoperative immobilization			
6 weeks hallux cast	259 (99.2%)	202 (78.0%)	57 (22.0%)
6 weeks short leg cast	1 (0.4%)	1 (100.0%)	0 (0.0%)
8-12 weeks hallux cast	1 (0.4%)	1 (100.0%)	0 (0.0%)
Shoe size (European standard)	40.4 (2.5)	40.3 (2.5)	40.6 (2.5)

Nonunion incidence was 26.9% in males compared to 20.1% in females. Average body weight was 82.7 kg (standard deviation [SD] 17.5) in the nonunion group vs. 78.6 kg (SD 16.1) in the union group (*P *= 0.104). Preoperative HVA was 31.4° (SD 14.6) in the nonunion group vs. 25.8° (SD 13.5) in the union group (*P *= 0.009). Postoperative HVA was 19.4° (SD 9.7) in the nonunion group vs. 16.2° (SD 8.0) in the union group (*P *= 0.013). Joint preparation with planar cuts had a nonunion incidence of 8.3% compared to 21.5% in convex/concave reaming and 25.5% when hand instruments were used. 

Influence of shoe size on nonunion

All variables were univariately tested for significance (Table 2). Only body weight (odds ratio [OR] 1.01, *P *= 0.104), preoperative HVA (OR 1.03, *P *= 0.009), postoperative HVA (OR 1.05, *P *= 0.013), and joint preparation with convex/concave reamers (OR 3.76, *P *= 0.086) were *P *< 0.157 and were, therefore, included in the multivariate regression analyses. 

**Table 2:** Univariate and multivariate logistic regression analysis estimating the effect of risk factors for nonunion. Differences were considered statistically significant with a *P*-value < 0.05 and are marked in bold. ^*^P < 0.157 and entered into the multiple regression analysis. *B*, nonstandardized regression coefficient; ASA, American Society of Anesthesiologists classification; BMI, body mass index; CC, convex/concave; IFS, interfragmentary screw; 95% CI, 95% confidence interval, lower-upper limit; OR, odds ratio

Variable	Univariate				Multivariate model 1				Multivariate model 2			
	B	OR	*P*-value	95% CI	B	OR	*P*-value	95% CI	B	OR	*P*-value	95% CI
Age, years	-0.007	0.99	0.615	0.97-1.02								
Sex (%) (ref = female)		1.46	0.250	0.77-2.78								
ASA (%) (ref = ≤ II)		1.96	0.293	0.56-6.84								
Side (%) (ref = right)		1.48	0.199	0.81-2.68								
BMI (kg/m^2^)	0.038	1.04	0.203	0.98-1.10								
Body weight (kg)	0.014	1.01	0.104^*^	1.00-1.03	0.02	1.02	0.143	1.00-1.04	0.02	1.02	0.177	0.99-1.04
Height (cm)	0.018	1.02	0.278	0.99-1.05								
Wound infection (ref = No)		1.64	0.428	0.49-5.52								
Additional forefoot surgery (ref = No)		1.19	0.667	0.54-2.60								
Hallux valgus angle (degrees)												
Preoperative	0.028	1.03	0.009^*^	1.01-1.05	0.03	1.03	0.008	1.01-1.05	0.02	1.02	0.204	0.99-1.05
Postoperative	0.044	1.05	0.013^*^	1.01-1.08					0.03	1.03	0.225	0.98-1.08
Joint preparation (ref = planar cuts)												
CC reaming		3.76	0.086^*^	0.83-17.11		3.43	0.114	0.75-15.80		3.33	0.123	0.72-15.34
Manual preparation		3.01	0.151^*^	0.67-13.55		2.35	0.274	0.51-10.90		2.30	0.288	0.47-10.67
Joint fixation (ref = plate + IFS)												
Crossed screws		1.12	0.921	0.12-10.22								
Plate only		1.00	1.000	0.08-12.56								
Postoperative immobilization (ref = 6 weeks hallux cast)												
6 weeks short leg cast		0.00	1.00	0.00-0.00								
8-12 weeks hallux cast		0.00	1.00	0.00-0.00								
Shoe size (European standard)	0.051	1.05	0.386	0.94-1.18	0.01	1.01	0.941	0.87-1.16	0.00	1.00	0.966	0.87-1.16

Shoe size was 41 (SD 2.5) in the nonunion group vs. 40 (SD 2.5) in the union group (*P *= 0.386). As self-reported shoe size was our primary outcome measure, it was also included in the multivariate models. However, no significance was found in either the univariate or the multivariate models (Table 2). 

Model 1 shows that preoperative HVA is the only factor associated with nonunion (OR 1.03, *P *= 0.008). After the inclusion of postoperative HVA in model 2, this effect diminishes.

## Discussion

Based on the results of this exploratory study, it can be concluded that foot size, operationalized using self-reported shoe size as a proxy, is not a risk factor for developing nonunion after the first MTPJ arthrodesis and as a result cannot be used as an explanation for the difference between men and women. Overall, we found 21.8% nonunion in 261 patients after primary first MTPJ arthrodesis, which is relatively high compared to other published literature [2,5].

The objective of this study was to elucidate the disparity in nonunion incidence observed between male and female subjects. While no distinction in shoe size is evident among the groups, it remains challenging for future research to establish the associations between actual foot size and other sex-related differences, and the risk of nonunion. Body weight and height did not show significant or clinically relevant differences between nonunion and union groups. Bass et al. hypothesized that the difference in nonunion frequency between the sexes was due to men’s lower adherence to postoperative instructions as they saw greater differences in nonlocking constructions. This hypothesis stands but only has a theoretical basis and will, therefore, need further investigation regarding postoperative loading, activity, and adherence to instructions [13].

Our choice of self-reported shoe size as a proxy for foot size can be criticized. Retail footwear comes in pairs with matching sizes. However, Kusumoto and Ashizawa uncovered that only 33% of their cohort had an equal left and right foot size [27]. Harrison et al. found a mere 24% of patients to be wearing correctly fitting shoes in terms of shoe length and width [28]. Many adults tend to stick to the shoe size they remember from their younger years when it was initially measured. However, foot size and shape change over time, influenced by age, activity, and various foot conditions [29, 30].

Using this exploratory study we wanted to obtain a first glimpse of the potential role of shoe size on nonunion; however, the outcome must be judged in the context of a few limitations. First, our exploration is based on retrospective data, consisting of patients who had undergone arthrodesis in the past. Also, patients with comorbidities were poorly registered, so the possible confounding effect of comorbidities on nonunion could not be determined. Since smoking is a risk factor for nonunion, we could not correct its possibly confounding effect [11,12]. However, this is the first study to investigate the association between shoe size as a proxy for foot size and nonunion after the first MTPJ arthrodesis. 

## Conclusions

Shoe size as a proxy for foot size does not have a clinical association with nonunion and as a result does not have to be considered when choosing a surgical technique or fixation for first MTPJ arthrodesis and thus does not explain the difference in nonunion incidence between males and females.
